# 4-Methyl-7-[2-(1*H*-1,2,4-triazol-1-yl)eth­oxy]-2*H*-chromen-2-one

**DOI:** 10.1107/S1600536811009159

**Published:** 2011-03-15

**Authors:** Yi-Yi Zhang, Yuan Shi, Cheng-He Zhou

**Affiliations:** a Laboratory of Bioorganic and Medicinal Chemistry, School of Chemistry and Chemical Engineering, Southwest University, Chongqing 400715, People’s Republic of China

## Abstract

In the title mol­ecule, C_14_H_13_N_3_O_3_, the dihedral angle between the triazole ring and coumarin ring system is 73.01 (4)°. The crystal structure is stabilized by weak inter­molecular C—H⋯N and C—H⋯O hydrogen bonds.

## Related literature

For the pharmacological activity of coumarins, see: Wu *et al.* (2009 ▶). For details of the synthesis, see: Shi & Zhou (2011 ▶).
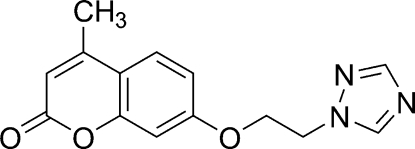


## Experimental

### 

#### Crystal data

C_14_H_13_N_3_O_3_
*M* *_r_* = 271.27
Monoclinic, 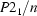

*a* = 11.9861 (17) Å
*b* = 7.7090 (11) Å
*c* = 14.132 (2) Å
β = 101.034 (2)°
*V* = 1281.7 (3) Å^3^
*Z* = 4
Mo *K*α radiation
μ = 0.10 mm^−1^
*T* = 173 K
0.40 × 0.30 × 0.24 mm

#### Data collection

Bruker SMART CCD diffractometer
6501 measured reflections
2390 independent reflections
2134 reflections with *I* > 2σ(*I*)
*R*_int_ = 0.025

#### Refinement

*R*[*F* ^2^ > 2σ(*F* ^2^)] = 0.037
*wR*(*F* ^2^) = 0.097
*S* = 1.04
2390 reflections
182 parameters
H-atom parameters constrained
Δρ_max_ = 0.16 e Å^−3^
Δρ_min_ = −0.23 e Å^−3^

### 

Data collection: *SMART* (Bruker, 2000 ▶); cell refinement: *SAINT* (Bruker, 2000 ▶); data reduction: *SAINT*; program(s) used to solve structure: *SHELXS97* (Sheldrick, 2008 ▶); program(s) used to refine structure: *SHELXL97* (Sheldrick, 2008 ▶); molecular graphics: *SHELXTL* (Sheldrick, 2008 ▶); software used to prepare material for publication: *SHELXTL*.

## Supplementary Material

Crystal structure: contains datablocks I, global. DOI: 10.1107/S1600536811009159/lh5217sup1.cif

Structure factors: contains datablocks I. DOI: 10.1107/S1600536811009159/lh5217Isup2.hkl

Additional supplementary materials:  crystallographic information; 3D view; checkCIF report

## Figures and Tables

**Table 1:** Hydrogen-bond geometry (Å, °)

*D*—H⋯*A*	*D*—H	H⋯*A*	*D*⋯*A*	*D*—H⋯*A*
C8—H8⋯N2^i^	0.95	2.56	3.453 (2)	157
C9—H9⋯N3^ii^	0.95	2.49	3.380 (2)	157
C13—H13⋯O1^iii^	0.95	2.48	3.408 (2)	165
C13—H13⋯O2^iii^	0.95	2.59	3.410 (2)	144
C14—H14⋯O3^iv^	0.95	2.55	3.481 (2)	166

Symmetry codes: (i) 
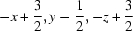
; (ii) 
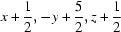
; (iii) 
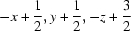
; (iv) 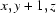.

**Fig. 1.**
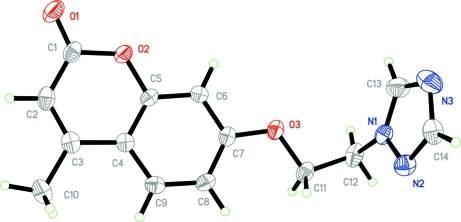
The molecular structure of (I) with displacement ellipsoids are drawn at the 50% probability level.

## supplementary crystallographic information

### Comment

Coumarins and their derivatives have attracted considerable attention due to their extensively biological activities such as antibacterial, antifungal, antiviral, anti-tubercular, anti-malarial, anticoagulant, anti-inflammatory, anticancer and antioxidant properties (Wu, *et al.*, 2009; Shi, *et al.*, 2011). In view of the therapeutic potentials of coumarins, we synthesized the title compound (I). Herein we report its crystal structure.

The molecular structure of the title compound is shown in Fig. 1. The dihedral angle between the triazole ring and coumarin ring system is 73.01 (4)°. The crystal structure is stabilized by weak intermolecular C—H···N and C—H···O hydrogen bonds.

### Experimental

Compound (I) was synthesized according to the procedure of Shi & Zhou (2011). Single crystals were grown by slow evaporation of a solution of (I) in CDCl_3_ at room temperature.

### Refinement

Hydrogen atoms were placed in idealized positions and treated as riding, with C—H = 0.95 Å (CH), 0.99 Å (CH_2_) *U*_iso_(H) = 1.2 *U*_eq_(CH, CH_2_) and 0.98 Å (CH_3_), *U*_iso_(H) = 1.5*U*_eq_(CH_3_).

### Crystal data

**Table:**

C_14_H_13_N_3_O_3_	*F*(000) = 568
*M**_r_* = 271.27	*D*_x_ = 1.406 Mg m^−^^3^
Monoclinic, *P*2_1_/*n*	Mo *K*α radiation, λ = 0.71073 Å
Hall symbol: -P 2yn	Cell parameters from 3660 reflections
*a* = 11.9861 (17) Å	θ = 2.5–28.1°
*b* = 7.7090 (11) Å	µ = 0.10 mm^−^^1^
*c* = 14.132 (2) Å	*T* = 173 K
β = 101.034 (2)°	Block, colourless
*V* = 1281.7 (3) Å^3^	0.40 × 0.30 × 0.24 mm
*Z* = 4	

### Data collection

**Table:**

Bruker SMART CCD diffractometer	2134 reflections with *I* > 2σ(*I*)
Radiation source: fine-focus sealed tube	*R*_int_ = 0.025
graphite	θ_max_ = 25.5°, θ_min_ = 2.0°
φ and ω scans	*h* = −14→14
6501 measured reflections	*k* = −9→9
2390 independent reflections	*l* = −17→14

### Refinement

**Table:**

Refinement on *F*^2^	Primary atom site location: structure-invariant direct methods
Least-squares matrix: full	Secondary atom site location: difference Fourier map
*R*[*F*^2^ > 2σ(*F*^2^)] = 0.037	Hydrogen site location: inferred from neighbouring sites
*wR*(*F*^2^) = 0.097	H-atom parameters constrained
*S* = 1.04	*w* = 1/[σ^2^(*F*_o_^2^) + (0.0489*P*)^2^ + 0.3468*P*] where *P* = (*F*_o_^2^ + 2*F*_c_^2^)/3
2390 reflections	(Δ/σ)_max_ < 0.001
182 parameters	Δρ_max_ = 0.16 e Å^−^^3^
0 restraints	Δρ_min_ = −0.23 e Å^−^^3^

### Special details

**Table:**

Geometry. All e.s.d.'s (except the e.s.d. in the dihedral angle between two l.s. planes) are estimated using the full covariance matrix. The cell e.s.d.'s are taken into account individually in the estimation of e.s.d.'s in distances, angles and torsion angles; correlations between e.s.d.'s in cell parameters are only used when they are defined by crystal symmetry. An approximate (isotropic) treatment of cell e.s.d.'s is used for estimating e.s.d.'s involving l.s. planes.
Refinement. Refinement of *F*^2^ against ALL reflections. The weighted *R*-factor *wR* and goodness of fit *S* are based on *F*^2^, conventional *R*-factors *R* are based on *F*, with *F* set to zero for negative *F*^2^. The threshold expression of *F*^2^ > σ(*F*^2^) is used only for calculating *R*-factors(gt) *etc*. and is not relevant to the choice of reflections for refinement. *R*-factors based on *F*^2^ are statistically about twice as large as those based on *F*, and *R*- factors based on ALL data will be even larger.

### Fractional atomic coordinates and isotropic or equivalent isotropic displacement parameters (Å^2^)

**Table:**

	*x*	*y*	*z*	*U*_iso_*/*U*_eq_	
C1	0.34092 (11)	0.63715 (16)	1.03382 (10)	0.0285 (3)	
C2	0.44570 (12)	0.61742 (16)	1.10231 (10)	0.0291 (3)	
H2	0.4448	0.5553	1.1602	0.035*	
C3	0.54494 (11)	0.68343 (16)	1.08755 (9)	0.0261 (3)	
C4	0.54683 (10)	0.77458 (15)	0.99847 (9)	0.0228 (3)	
C5	0.44581 (10)	0.78927 (15)	0.93081 (9)	0.0222 (3)	
C6	0.43918 (10)	0.87313 (15)	0.84385 (9)	0.0233 (3)	
H6	0.3692	0.8796	0.7992	0.028*	
C7	0.53705 (11)	0.94803 (15)	0.82283 (9)	0.0232 (3)	
C8	0.64008 (10)	0.93534 (16)	0.88806 (10)	0.0258 (3)	
H8	0.7071	0.9852	0.8732	0.031*	
C9	0.64365 (11)	0.84983 (16)	0.97420 (10)	0.0258 (3)	
H9	0.7140	0.8418	1.0184	0.031*	
C10	0.65229 (12)	0.66473 (19)	1.16165 (10)	0.0351 (3)	
H10A	0.6375	0.5922	1.2149	0.053*	
H10B	0.6783	0.7795	1.1864	0.053*	
H10C	0.7111	0.6103	1.1320	0.053*	
C11	0.62329 (11)	1.10015 (17)	0.70725 (10)	0.0296 (3)	
H11A	0.6776	1.0054	0.7024	0.036*	
H11B	0.6612	1.1859	0.7549	0.036*	
C12	0.58548 (13)	1.18489 (17)	0.61091 (10)	0.0326 (3)	
H12A	0.6525	1.2038	0.5807	0.039*	
H12B	0.5330	1.1058	0.5687	0.039*	
C13	0.42086 (12)	1.3931 (2)	0.58744 (11)	0.0379 (4)	
H13	0.3631	1.3152	0.5581	0.046*	
C14	0.50929 (12)	1.61203 (18)	0.64494 (10)	0.0328 (3)	
H14	0.5252	1.7284	0.6651	0.039*	
N1	0.52866 (9)	1.35020 (14)	0.61733 (8)	0.0256 (3)	
N2	0.58838 (9)	1.49167 (14)	0.65533 (8)	0.0308 (3)	
N3	0.40436 (11)	1.55923 (18)	0.60384 (10)	0.0440 (3)	
O1	0.24796 (9)	0.58794 (14)	1.04329 (8)	0.0415 (3)	
O2	0.34566 (7)	0.71985 (11)	0.94832 (6)	0.0267 (2)	
O3	0.52351 (8)	1.03232 (12)	0.73646 (6)	0.0278 (2)	

### Atomic displacement parameters (Å^2^)

**Table:**

	*U*^11^	*U*^22^	*U*^33^	*U*^12^	*U*^13^	*U*^23^
C1	0.0340 (7)	0.0207 (6)	0.0344 (8)	−0.0007 (5)	0.0152 (6)	0.0017 (5)
C2	0.0418 (8)	0.0215 (6)	0.0263 (7)	0.0029 (5)	0.0123 (6)	0.0015 (5)
C3	0.0347 (7)	0.0188 (6)	0.0253 (7)	0.0038 (5)	0.0067 (5)	−0.0041 (5)
C4	0.0271 (6)	0.0177 (6)	0.0240 (6)	0.0019 (5)	0.0060 (5)	−0.0036 (5)
C5	0.0232 (6)	0.0173 (6)	0.0281 (7)	−0.0012 (5)	0.0097 (5)	−0.0029 (5)
C6	0.0228 (6)	0.0220 (6)	0.0247 (7)	0.0005 (5)	0.0035 (5)	−0.0021 (5)
C7	0.0290 (6)	0.0181 (6)	0.0243 (7)	0.0014 (5)	0.0094 (5)	−0.0020 (5)
C8	0.0227 (6)	0.0231 (6)	0.0335 (7)	−0.0014 (5)	0.0100 (5)	−0.0023 (5)
C9	0.0233 (6)	0.0229 (6)	0.0302 (7)	0.0017 (5)	0.0031 (5)	−0.0035 (5)
C10	0.0429 (8)	0.0334 (8)	0.0268 (7)	0.0034 (6)	0.0009 (6)	0.0019 (6)
C11	0.0320 (7)	0.0241 (6)	0.0371 (8)	0.0015 (5)	0.0175 (6)	0.0023 (6)
C12	0.0465 (8)	0.0242 (7)	0.0321 (8)	0.0010 (6)	0.0205 (6)	−0.0015 (6)
C13	0.0297 (7)	0.0448 (9)	0.0377 (8)	−0.0036 (6)	0.0025 (6)	−0.0003 (7)
C14	0.0435 (8)	0.0268 (7)	0.0289 (7)	0.0043 (6)	0.0090 (6)	−0.0007 (6)
N1	0.0294 (6)	0.0252 (5)	0.0238 (6)	−0.0027 (4)	0.0093 (4)	−0.0006 (4)
N2	0.0309 (6)	0.0274 (6)	0.0341 (6)	−0.0019 (5)	0.0061 (5)	−0.0039 (5)
N3	0.0365 (7)	0.0479 (8)	0.0460 (8)	0.0129 (6)	0.0040 (6)	0.0032 (6)
O1	0.0359 (6)	0.0416 (6)	0.0514 (7)	−0.0049 (5)	0.0192 (5)	0.0119 (5)
O2	0.0243 (5)	0.0264 (5)	0.0305 (5)	−0.0035 (4)	0.0083 (4)	0.0031 (4)
O3	0.0305 (5)	0.0279 (5)	0.0266 (5)	−0.0017 (4)	0.0094 (4)	0.0039 (4)

### Geometric parameters (Å, °)

**Table:**

C1—O1	1.2085 (16)	C10—H10A	0.9800
C1—O2	1.3769 (16)	C10—H10B	0.9800
C1—C2	1.439 (2)	C10—H10C	0.9800
C2—C3	1.3463 (19)	C11—O3	1.4364 (15)
C2—H2	0.9500	C11—C12	1.500 (2)
C3—C4	1.4457 (18)	C11—H11A	0.9900
C3—C10	1.5023 (19)	C11—H11B	0.9900
C4—C5	1.3964 (18)	C12—N1	1.4560 (17)
C4—C9	1.3975 (17)	C12—H12A	0.9900
C5—C6	1.3774 (18)	C12—H12B	0.9900
C5—O2	1.3793 (14)	C13—N1	1.3223 (18)
C6—C7	1.3896 (17)	C13—N3	1.323 (2)
C6—H6	0.9500	C13—H13	0.9500
C7—O3	1.3647 (15)	C14—N2	1.3145 (17)
C7—C8	1.3960 (18)	C14—N3	1.344 (2)
C8—C9	1.3778 (19)	C14—H14	0.9500
C8—H8	0.9500	N1—N2	1.3576 (15)
C9—H9	0.9500		
			
O1—C1—O2	115.86 (12)	H10A—C10—H10B	109.5
O1—C1—C2	126.63 (13)	C3—C10—H10C	109.5
O2—C1—C2	117.50 (11)	H10A—C10—H10C	109.5
C3—C2—C1	122.62 (12)	H10B—C10—H10C	109.5
C3—C2—H2	118.7	O3—C11—C12	107.20 (11)
C1—C2—H2	118.7	O3—C11—H11A	110.3
C2—C3—C4	118.69 (12)	C12—C11—H11A	110.3
C2—C3—C10	121.34 (13)	O3—C11—H11B	110.3
C4—C3—C10	119.97 (12)	C12—C11—H11B	110.3
C5—C4—C9	116.76 (12)	H11A—C11—H11B	108.5
C5—C4—C3	118.66 (11)	N1—C12—C11	112.86 (11)
C9—C4—C3	124.58 (12)	N1—C12—H12A	109.0
C6—C5—O2	116.01 (11)	C11—C12—H12A	109.0
C6—C5—C4	122.92 (11)	N1—C12—H12B	109.0
O2—C5—C4	121.07 (11)	C11—C12—H12B	109.0
C5—C6—C7	118.60 (11)	H12A—C12—H12B	107.8
C5—C6—H6	120.7	N1—C13—N3	110.86 (13)
C7—C6—H6	120.7	N1—C13—H13	124.6
O3—C7—C6	115.36 (11)	N3—C13—H13	124.6
O3—C7—C8	124.24 (11)	N2—C14—N3	115.46 (13)
C6—C7—C8	120.40 (12)	N2—C14—H14	122.3
C9—C8—C7	119.40 (11)	N3—C14—H14	122.3
C9—C8—H8	120.3	C13—N1—N2	109.53 (11)
C7—C8—H8	120.3	C13—N1—C12	129.74 (12)
C8—C9—C4	121.91 (12)	N2—N1—C12	120.68 (11)
C8—C9—H9	119.0	C14—N2—N1	102.03 (11)
C4—C9—H9	119.0	C13—N3—C14	102.12 (12)
C3—C10—H10A	109.5	C1—O2—C5	121.37 (10)
C3—C10—H10B	109.5	C7—O3—C11	117.86 (10)
			
O1—C1—C2—C3	−176.91 (13)	C5—C4—C9—C8	0.34 (18)
O2—C1—C2—C3	3.28 (19)	C3—C4—C9—C8	−179.93 (11)
C1—C2—C3—C4	−1.36 (19)	O3—C11—C12—N1	74.23 (14)
C1—C2—C3—C10	178.33 (12)	N3—C13—N1—N2	−0.17 (17)
C2—C3—C4—C5	−0.56 (17)	N3—C13—N1—C12	−177.64 (13)
C10—C3—C4—C5	179.75 (11)	C11—C12—N1—C13	−111.33 (16)
C2—C3—C4—C9	179.72 (12)	C11—C12—N1—N2	71.44 (15)
C10—C3—C4—C9	0.02 (18)	N3—C14—N2—N1	0.17 (16)
C9—C4—C5—C6	−0.06 (18)	C13—N1—N2—C14	0.00 (15)
C3—C4—C5—C6	−179.80 (11)	C12—N1—N2—C14	177.75 (11)
C9—C4—C5—O2	−179.74 (10)	N1—C13—N3—C14	0.25 (16)
C3—C4—C5—O2	0.51 (17)	N2—C14—N3—C13	−0.26 (17)
O2—C5—C6—C7	179.02 (10)	O1—C1—O2—C5	176.87 (11)
C4—C5—C6—C7	−0.68 (18)	C2—C1—O2—C5	−3.29 (17)
C5—C6—C7—O3	−178.52 (10)	C6—C5—O2—C1	−178.20 (11)
C5—C6—C7—C8	1.15 (18)	C4—C5—O2—C1	1.50 (17)
O3—C7—C8—C9	178.75 (11)	C6—C7—O3—C11	−175.04 (10)
C6—C7—C8—C9	−0.89 (18)	C8—C7—O3—C11	5.30 (17)
C7—C8—C9—C4	0.13 (19)	C12—C11—O3—C7	179.81 (10)

### Hydrogen-bond geometry (Å, °)

**Table:**

*D*—H···*A*	*D*—H	H···*A*	*D*···*A*	*D*—H···*A*
C8—H8···N2^i^	0.95	2.56	3.453 (2)	157
C9—H9···N3^ii^	0.95	2.49	3.380 (2)	157
C13—H13···O1^iii^	0.95	2.48	3.408 (2)	165
C13—H13···O2^iii^	0.95	2.59	3.410 (2)	144
C14—H14···O3^iv^	0.95	2.55	3.481 (2)	166

Symmetry codes: (i) −*x*+3/2, *y*−1/2, −*z*+3/2; (ii) *x*+1/2, −*y*+5/2, *z*+1/2; (iii) −*x*+1/2, *y*+1/2, −*z*+3/2; (iv) *x*, *y*+1, *z*.
